# *α*-Glucosidase Inhibitory Activity and Anti-Adipogenic Effect of Compounds from *Dendrobium delacourii*

**DOI:** 10.3390/molecules27041156

**Published:** 2022-02-09

**Authors:** May Thazin Thant, Hnin Ei Ei Khine, Justin Quiel Lasam Nealiga, Nutputsorn Chatsumpun, Chatchai Chaotham, Boonchoo Sritularak, Kittisak Likhitwitayawuid

**Affiliations:** 1 Department of Pharmacognosy and Pharmaceutical Botany, Faculty of Pharmaceutical Sciences, Chulalongkorn University, Bangkok 10330, Thailand; drmaythazinthant@gmail.com (M.T.T.); kittisak.l@chula.ac.th (K.L.); 2 Department of Pharmacognosy, University of Pharmacy, Yangon 11031, Myanmar; 3 Department of Biochemistry and Microbiology, Faculty of Pharmaceutical Sciences, Chulalongkorn University, Bangkok 10330, Thailand; hnineieikhine12@gmail.com (H.E.E.K.); jquieln@gmail.com (J.Q.L.N.); 4 Department of Pharmacognosy, Faculty of Pharmacy, Mahidol University, Bangkok 10400, Thailand; nutputsorn.cha@mahidol.ac.th; 5 Preclinical Toxicity and Efficacy Assessment of Medicines and Chemicals Research Unit, Faculty of Pharmaceutical Sciences, Chulalongkorn University, Bangkok 10330, Thailand; 6 Natural Products for Ageing and Chronic Diseases Research Unit, Faculty of Pharmaceutical Sciences, Chulalongkorn University, Bangkok 10330, Thailand

**Keywords:** *Dendrobium delacourii*, Orchidaceae, *α*-glucosidase, anti-adipogenic, densifloral B, phoyunnanin E, phoyunnanin C

## Abstract

Chemical investigation of *Dendrobium delacourii* revealed 11 phenolic compounds, and the structures of these compounds were determined by analysis of their NMR and HR-ESI-MS data. All compounds were investigated for their *α*-glucosidase inhibitory activity and anti-adipogenic properties. Phoyunnanin E (**10**) and phoyunnanin C (**11**) showed the most potent *α*-glucosidase inhibition by comparing with acarbose, which was used as a positive control. Kinetic study revealed the non-competitive inhibitors against the enzyme. For anti-adipogenic activity, densifloral B (**3**) showed the strongest inhibition when compared with oxyresveratrol (positive control). In addition, densifloral B might be responsible for the inhibition of adipocyte differentiation via downregulating the expression of peroxisome proliferator-activated receptor gamma (PPARγ) and CCAAT enhancer-binding protein alpha (C/EBP*α*), which are major transcription factors in adipogenesis.

## 1. Introduction

Diabetes mellitus (DM) is a chronic metabolic disorder characterized by a high level of blood glucose resulting from a relative or absolute deficiency of insulin action. Type II is the most common type of diabetes, caused by *β*-cell dysfunction and insulin resistance [1]. The inhibition of *α*-glucosidase is effective for the treatment of type II diabetes [2]. *α*-Glucosidase is a membrane-bound enzyme produced from the epithelial cells of the small intestine. This enzyme is capable of converting starch and disaccharides into monosaccharides (glucose). Thus, glucose absorption can be reduced by inhibition of this enzyme, and postprandial blood glucose levels can also be decreased [3,4].

The relation between diabetes and obesity is well established in both traditional and modern therapy. The World Health Organization (WHO) estimates that 44% of diabetes cases are associated with overweightness and obesity [5]. Obesity is a risk factor of type 2 diabetes, coronary heart disease, and hypertension and is becoming a major health problem [6]. As a complex multifactorial chronic disease, obesity is characterized by an excessive adipocyte tissue mass. Adipogenesis is the process of cell differentiation during which fibroblast-like preadipocytes develop into mature adipocytes. Recently, the inhibition of adipogenesis has been proposed as a promising anti-obesity approach [7]. Moreover, anti-obesity is able considerably to reduce the prevalence rate of type 2 diabetes [8].

Currently, drugs from natural sources that specifically inhibit peroxisome proliferator-activated receptor gamma (PPAR*γ*) and CCAAT enhancer-binding protein alpha (C/EBP*α*) expression are being targeted for the treatment of obesity [9]. PPAR*γ* and C/EBP*α* increase adipocyte differentiation by activating the gene transcription for generating the adipocyte phenotype [10]. The anti-adipogenic activities of several natural compounds such as catechin [11] and procyanidin [12] via the downregulation of C/EBP*α* and PPAR*γ* have been reported. Moreover, AMP-activated protein kinase (AMPK) is a common regulator that is involved in various lipid metabolisms [13], and the regulation of the AMPK signaling pathway is essential for anti-obesity [14]. The activation of AMPK can downregulate adipogenic key transcription factors such as PPAR*γ* and C/EBP*α*, resulting in the suppression of adipocyte differentiation [15]. Acetyl-CoA carboxylase (ACC) is also responsible for the reduction in fatty acid synthesis [16]; in other words, fat accumulation is inhibited by the activation of ACC [17,18]. Adipogenesis can be inhibited by increasing the phosphorylation of both AMPK and ACC [19]. Protein kinase B (Akt) and glycogen synthase kinase-3 beta (GSK3*β*) are important protein kinases in adipogenesis. The activation of Akt is associated with phosphorylated GSK3*β* triggering adipogenesis via upregulating C/EBP*α* and PPAR*γ* [20,21].

The drugs currently available for the treatment of diabetes and obesity are accompanied by severe adverse effects such as insomnia, headache, hypoglycemia, weight gain, constipation, and renal damage [22,23]. There are increasing reports of natural drugs from plant sources, due to their lesser side-effects. *Dendrobium* species are widely used in traditional medicine for the treatment of various diseases, as they possess a variety of pharmacological properties, such as being antidiabetic, antioxidant, anti-inflammatory, antimicrobial, immunomodulatory, and anticancer [24]. In China, *Dendrobium* has been used as traditional medicine for thousands of years as source of tonic, astringent, analgesic, antipyretic, and anti-inflammatory action [25,26]. About 41 species of the *Dendrobium* genus have been recorded in traditional Chinese medicine (TCM); of these, 30 species are collectively known under the Chinese name ‘shihu’ and are used for nourishing the stomach and increasing bodily fluid production [27]. Some species of this genus have been used in Thai traditional medicine, including *D. cumulatum*, *D. draconis*, *D. indivisum*, *D. trigonopus*, and *D. leonis* [28].

**Figure 1:**
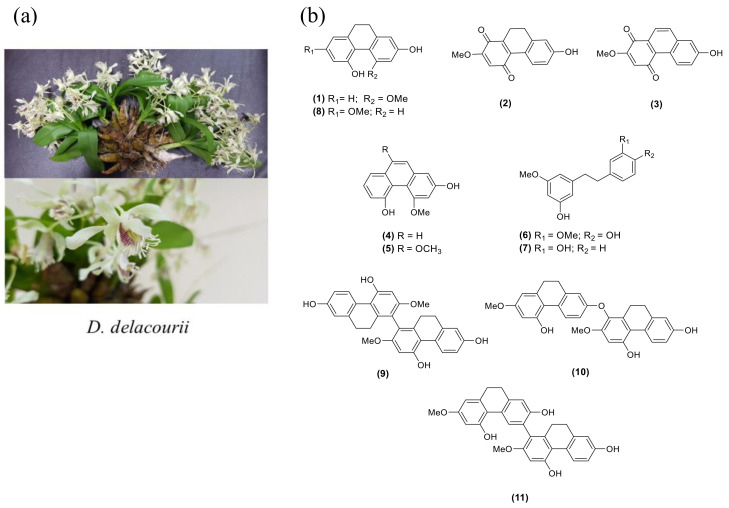
(**a**) *Dendrobium delacourii*. (**b**) The structures of isolated compounds (**1**–**11**).

*Dendrobium delacourii*, named ‘Ueang Dok Ma Kham’ in Thai, belongs to the family Orchidaceae (Figure 1a). The common name is Delacour’s dendrobium, and the plant is native to Thailand, Vietnam, Laos, and Myanmar. There is no previous study on the phytochemical constituents and biological activities of this plant. Our screening of the methanolic extract of *D. delacourii* exhibited *α*-glucosidase inhibition (80% inhibition at 100 μg/mL). It also showed an inhibitory effect on adipocyte differentiation in 3T3-L1 cells (51% inhibition at 5 μg/mL). In this study, we report the phytochemical constituents of *D. delacourii*, along with their inhibitory effects on *α*-glucosidase and adipocyte differentiation.

## 2. Results and Discussion

### 2.1. Structure Determination

A MeOH extract of *D. delacourii* was separated by solvent partition to give ethyl acetate, butanol, and aqueous extracts. These extracts were evaluated for their inhibition of the *α*-glucosidase enzyme and adipocyte differentiation of 3T3-L1 cells. Only the EtOAc extract showed potent inhibition of *α*-glucosidase (85.5% inhibition at 100 μg/mL) and an anti-adipogenic effect (49% inhibition at 5 μg/mL). Therefore, the EtOAc extract was selected for further phytochemical investigation. Chromatographic separation of the EtOAc extract resulted in the isolation of 11 compounds. The structures of isolated compounds were characterized through analysis of their spectroscopic data and in comparison with previous reported values and were identified as hircinol (**1**) [29], ephemeranthoquinone (**2**) [30], densifloral B (**3**) [31], moscatin (**4**) [32], 4,9-dimethoxy-2,5-phenanthrenediol (**5**) [33], gigantol (**6**) [34], batatasin III (**7**) [35], lusianthridin (**8**) [36], 4,4′,7,7′-tetrahydroxy-2,2′-dimethoxy-9,9′,10,10′-tetrahydro-1,1′-biphenanthrene (**9**) [36], phoyunnanin E (**10**) [36], and phoyunnanin C (**11**) [37] (Figure 1b).

### 2.2. α-Glucosidase Inhibitory Activity

All the isolated compounds (**1**–**11**) were evaluated for their *α*-glucosidase inhibitory activity. In this study, each compound was initially tested at 100 μg/mL. An IC_50_ was determined if the compound showed more than 50% inhibition of the enzyme. The results are summarized in Table 1. Moscatin (**4**), gigantol (**6**), and lusianthridin (**8**) showed moderate *α*-glucosidase inhibition, having IC_50_ values of 390.1 ± 9.8 μM, 191.3 ± 6.8 μM, and 195.4 ± 9.6 μM, respectively. In addition, 4,4′,7,7′-tetrahydroxy-2,2′-dimethoxy-9,9′,10,10′-tetrahydro-1,1′-biphenanthrene (**9**), phoyunnanin E (**10**), and phoyunnanin C (**11**) showed stronger *α*-glucosidase inhibitory activities, with IC_50_ values of 18.4 ± 3.4 μM, 8.9 ± 0.8 μM, and 12.6 ± 0.9 μM, respectively, as compared with the positive control acarbose (IC_50_ 514.4 ± 9.2 μM). It can be observed herein that the dimeric phenanthrene derivatives **9**, **10**, and **11** were more potent than the monomers **4** and **8**.

**Figure 2:**
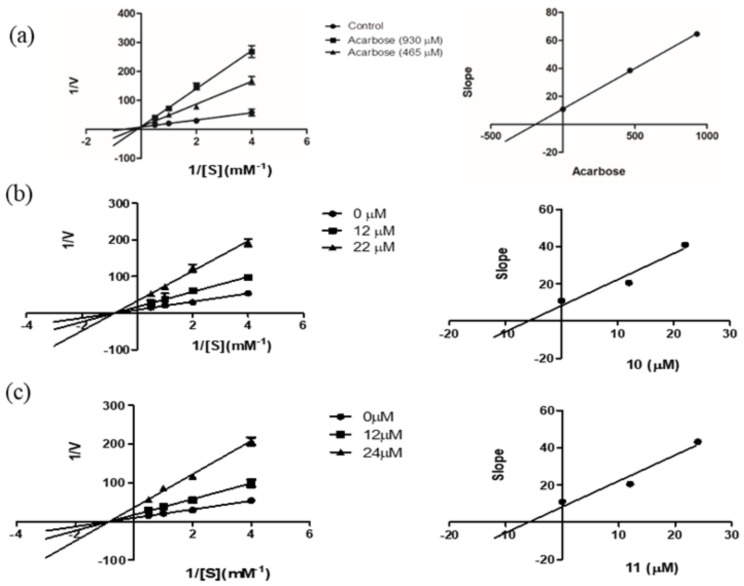
Lineweaver–Burk plots of (**a**) acarbose, (**b**) phoyunnanin E (**10**), and (**c**) phoyunnanin C (**11**). The secondary plot of each compound is on the right.

For further investigation of the mechanism of enzyme inhibition, a kinetic study was carried out on the most potent compounds, phoyunnanin E (**10**) and phoyunnanin C (**11**). The experiment was performed by using Lineweaver–Burk plots of the reciprocal of velocity (1/V) against the reciprocal of substrate concentration (1/[S]) (Figure 2). The substrate *p*-nitrophenol-*α*-D-glucopyranoside concentration was varied from 0.25 to 2.0 mM in the absence or presence of compound **10** at 12 μM and 22 μM and compound **11** at 12 μM and 24 μM. As summarized in Table 2, the different concentrations of **10** and **11** reduced the V*_max_* but did not affect the K*_m_* value, indicating that **10** and **11** are non-competitive types of enzyme inhibitors. On the other hand, the drug acarbose showed an intersection of the lines on the *y*-axis, indicating a competitive type of inhibition. A secondary plot of each compound was then constructed to evaluate the inhibition constant (K*_i_*). We found that the K*_i_* value of acarbose 190.57 μM was obtained, and both **10** (K*_i_* 5.89 μM) and **11** (K*_i_* 5.97 μM) showed much greater affinity to the enzyme than acarbose. Compounds **10** and **11** as non-competitive inhibitors have some benefit over competitive inhibitors according to their binding to the allosteric site of the enzyme; therefore, they do not depend on the substrate concentration [38]. Furthermore, non-competitive inhibitors demand lower concentrations than competitive inhibitors to generate the same result [39].

### 2.3. Anti-Adipogenic Activity

#### 2.3.1. Ethyl Acetate Extracts from *D. delacourii* Attenuate Lipid Accumulation in Differentiated Adipocytes

**Figure 3:**
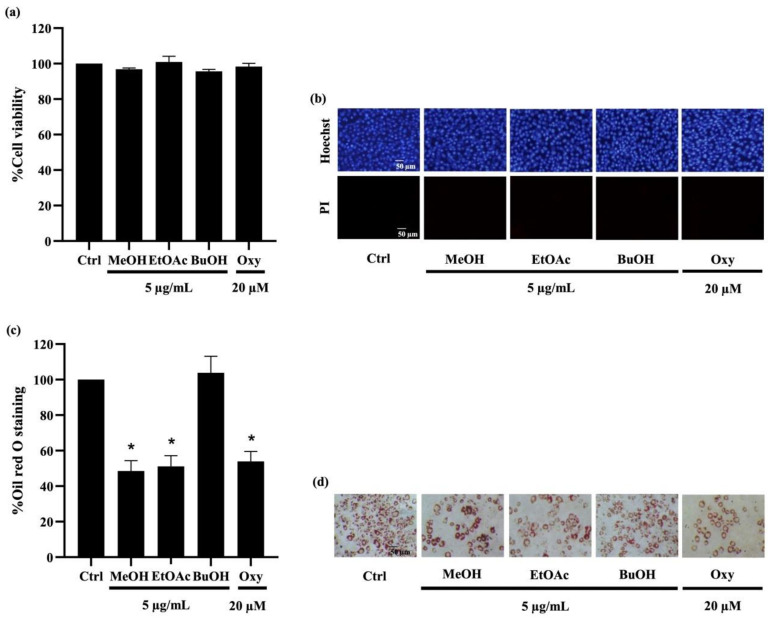
Effect of extracts from *Dendrobium delacourii* on cell viability and intracellular lipid accumulation during adipocyte differentiation. (**a**) MTT viability assay revealed no alteration of cell viability percentage in preadipocyte 3T3-L1 cells cultured with 5 µg/mL of methanolic extract (MeOH), ethyl acetate extract (EtOAc), or butanolic extract (BuOH) from *D. delacourii* for 48 h. (**b**) Treatment with all *D. delacourii* extracts for 48 h did not cause apoptosis or necrosis cell death detected via costaining of Hoechst33342/propidium iodide (PI) in 3T3-L1 cells. The suppression of lipid accumulation during adipogenesis in preadipocyte 3T3-L1 cells incubated with 5 µg/mL MeOH, 5 µg/mL EtOAc or 20 µM oxyresveratrol (Oxy) as a positive control was evidenced with (**c**) lower oil red O staining percentage and (**d**) lower amount of lipid droplets containing cells stained by oil red O compared with untreated control (Ctrl) group. Data are presented as means ± SD from three independent experiments. * *p* < 0.05 versus non-treated control cells.

To investigate their anti-adipogenic effect, the cytotoxic profile of *D. delacourii* extracts in preadipocytes was determined using MTT (methyl-thiazolyl-diphenyl-tetrazolium bromide) and nuclear staining assays. After culturing with 5 µg/mL of the methanolic, ethyl acetate, or butanolic extracts for 48 h, there were no significant alterations in the viability percentage observed via MTT assay in mouse embryonic preadipocyte 3T3-L1 cells (Figure 3a), compared with the untreated control. It is worth noting that treatment with all extracts at 10–20 µg/mL reduced viability in 3T3-L1 cells to lower than 90% (data not shown). Figure 3b depicts neither apoptosis nor necrosis, which were respectively observed as bright blue fluorescence of Hoechst33342 and propidium iodide red fluorescence in all treated 3T3-L1 cells. Thus, the extracts at 5 µg/mL, which were considered as non-toxic concentration, were chosen for the investigation of anti-adipogenic activity. Notably, treatment with oxyresveratrol (positive control), an anti-adipogenic natural compound, at 20 µM for 48 h also caused no change in cell viability percentage and cell death in preadipocyte 3T3-L1 cells.

For determination of their anti-adipogenic effect, preadipocyte 3T3-L1 cells were incubated with the differentiation medium with or without *D. delacourii* extracts, as mentioned in Materials and Methods. After the differentiation period was complete, the accumulated intracellular lipid droplets were analyzed by oil red O staining. As shown in Figure 3c, the lower level of oil red O percentage was indicated in 3T3-L1 cells cultured either with 5 µg/mL methanolic extract or 5 µg/mL ethyl acetate extract, compared with the control group. Meanwhile the significant decrease in oil red O staining percentage was not demonstrated in the treatment of the butanolic extract. A comparable inhibition of adipocyte differentiation between the methanolic extract, the ethyl acetate extract, and oxyresveratrol (20 µM) was evidenced with not only the reduction in the oil red O percentage but also the diminution of cellular lipid droplets presented in differentiated 3T3-L1 cells stained with oil red O (Figure 3d). Taken together, the ethyl acetate extract that indicated anti-adipogenic potential was selected for further investigation.

#### 2.3.2. Screening for Anti-Adipogenic Activity of Compounds (**1**–**11**)

**Figure 4:**
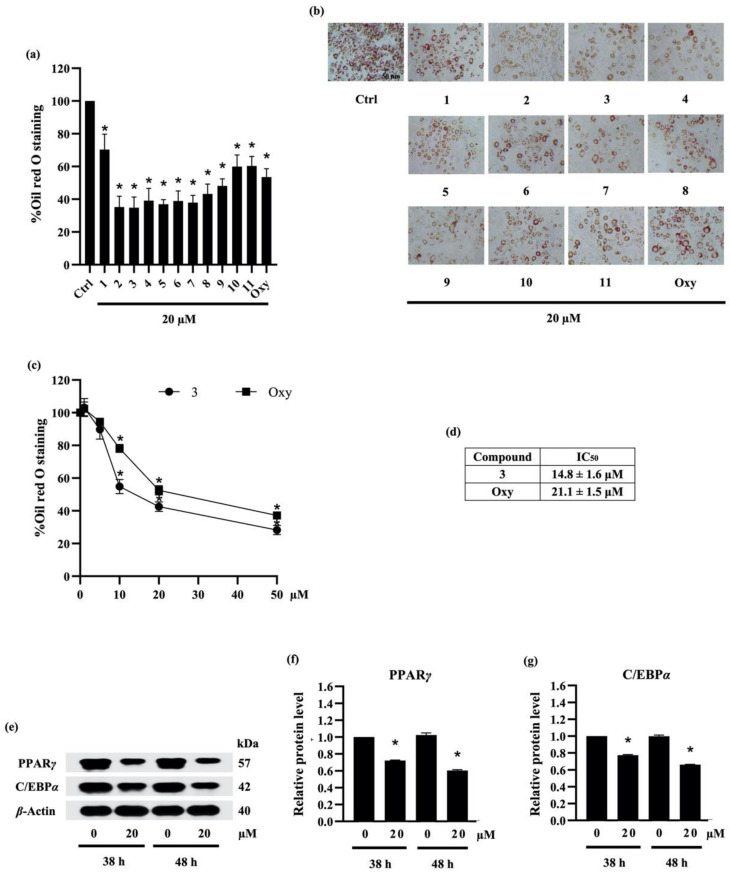
The effect of compounds (**1**–**11**) from *Dendrobium delacourii* on lipid accumulation in differentiated adipocyte was indicated with (**a**) the percentage of oil red O staining and (**b**) the accumulation of lipid droplets detected by oil red O staining. In comparison with a positive control, oxyresveratrol (Oxy), (**c**) dose–response relationship and (**d**) half-maximum inhibitory concentration (IC_50_) demonstrated the more potent anti-adipogenic activity of compound **3** in 3T3-L1 cells. (**e**) Immunoblot analysis revealed the decreased protein levels of key lipid transcriptional regulators in 3T3-L1 cells cultured with 20 µM compound **3** for both early (38 h) and late (48 h) time points. The significant reduction in (**f**) PPAR*γ* and (**g**) C/EBP*α* presented early in preadipocyte 3T3-L1 cells after the incubation with 20 µM compound **3** for 38 h in comparison with the no treatment control (Ctrl) at the same time point. *β*-actin was used as an internal control. Data are presented as means ± SD from three independent experiments. * *p* < 0.05 versus non-treated control cells at the same time point.

According to previous report about anti-adipogenic activity of phenolic compound, batatasin I, at 20 μM [40], the suppressive effect of compounds (**1**–**11**) isolated from ethyl acetate extract of *D. delacourii* at the same concentration (20 µM) was preliminarily demonstrated by the remarkable reduction in both oil red O staining percentage (Figure 4a) and oil red O staining cells (Figure 4b) in differentiated 3T3-L1 cells. Although ephemeranthoquinone (**2**) and densifloral B (**3**) showed the highest anti-adipogenic activity among various isolates as well as positive control (oxyresveratrol), with approximately 64.7% and 65.2% reduction in oil red O staining, respectively, compound **2** at 20 µM significantly decreased viability in 3T3-L1 cells (data not shown). Therefore, compound **3**, which caused no cytotoxicity in 3T3-L1 cells, was selected to further investigate the related anti-adipogenic mechanisms.

To compare the potency with another natural compound, preadipocyte 3T3-L1 cells were cultured with differentiation medium containing either various concentrations (0–50 µM) of compound **3** or oxyresveratrol. Figure 4c presents the relationship between concentration and oil red O staining percentage in response to compound **3** and oxyresveratrol treatment. The results indicate that compound **3** and oxyresveratrol clearly restrained the adipocyte differentiation in 3T3-L1 cells in a dose-dependent manner. In addition, the effect of a 50% inhibitory concentration (IC_50_) of compound **3** and oxyresveratrol on adipocyte differentiation was about 14.8 ± 1.6 µM and 21.1 ± 1.5 µM, respectively (Figure 4d). According to IC_50_ data, compound **3** showed higher potency in anti-adipogenesis compared with oxyresveratrol.

#### 2.3.3. Densifloral B (**3**) Suppresses Adipocyte Differentiation-Related Proteins

Adipogenesis involves a network of transcription factors that contribute to adipocyte differentiation and lipid accumulation [41]. Although the adipogenic gene CCAAT-enhancer-binding protein beta (C/EBP*β*) is expressed soon after exposure to the adipogenic inducers, the upregulation of PPAR*γ* and C/EBP*α* is acquired after 36–48 h of the induction [42]. In this regard, the translational level of PPAR*γ* and C/EBP*α* was examined to elucidate the anti-adipogenic mechanism in preadipocyte 3T3-L1 cells cultured with 20 µM densifloral B (**3**) at both early (38 h) and late (48 h) stage. Western blot analysis indicated the prolong expression of PPAR*γ* and C/EBP*α*, the adipogenic transcription factors, in 3T3-L1 cells from early until late stage of adipogenic process. Moreover, the expression level of PPAR*γ* and C/EBP*α* was restrained in densifloral B-treated 3T3-L1 cells compared with untreated control cells (Figure 4e). Interestingly, the diminution in PPAR*γ* and C/EBP*α* relative protein levels, which was promptly detected at 38 h, was sustained until 48 h of adipocyte differentiation in the presence of 20 µM densifloral B (Figure 4f,g, respectively).

**Figure 5:**
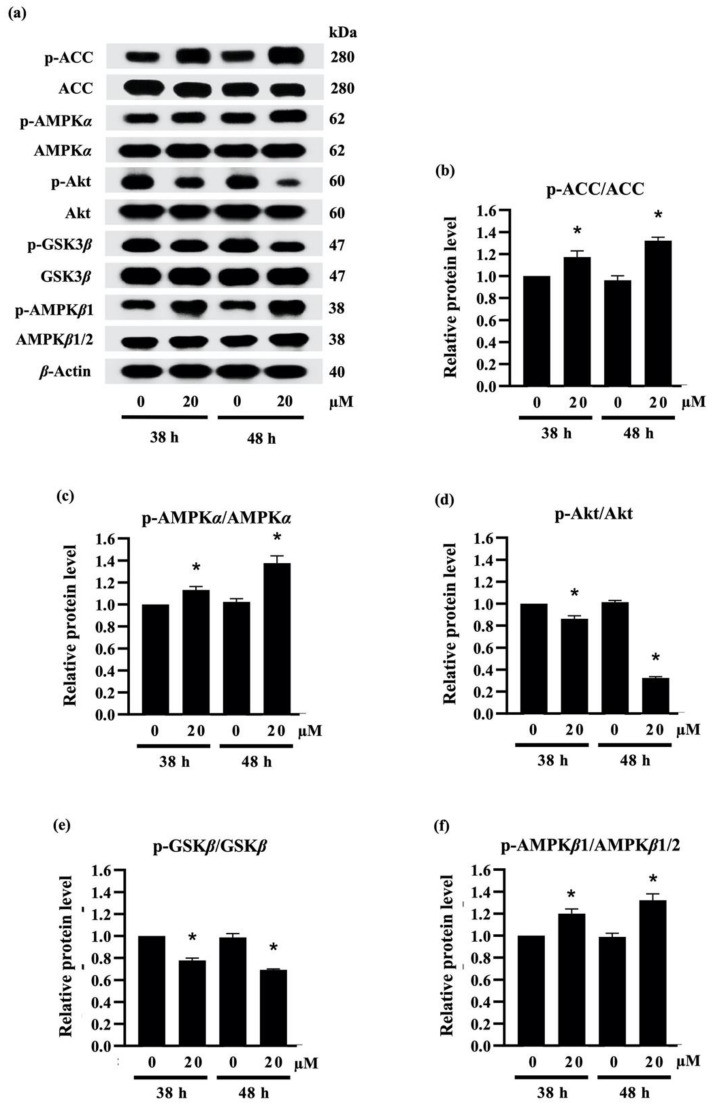
Effect of densifloral B (**3**) on adipocyte differentiation via Akt-related pathways (**a**) The alteration of adipogenesis-related proteins was determined in preadipocyte 3T3-L1 cells after culture with differentiation medium containing 0–20 µM densifloral B for 38–48 h via Western blotting. The upregulated levels of (**b**) p-ACC/ACC, (**c**) p-AMPK*α*/AMPK*α*, and (**f**) p-AMPK*β*1/AMPK*β*1/2, as well as the reduction in (**d**) pAkt/Akt and (**e**) p-GSK3*β*/GSK3*β*, were obviously presented in densifloral B-treated 3T3-L1 cells. *β*-actin served as an internal control. Data are presented as means ± SD from three independent experiments. * *p* < 0.05 versus non-treated control cells at the same time point.

Recent reports indicate that the downregulation of PPAR*γ* and C/EBP*α* are correlated with the activation of AMPK*α*/*β* and ACC [43]. As presented in Figure 5a, treatment with 20 µM densifloral B (**3**) upregulated the phosphorylated form of AMPK*α* (p-AMPK*α*), AMPK*β*1 (p-AMPK*β*1), and ACC (p-ACC) in 3T3-L1 cells at both 38 h and 48 h of the differentiation period. The increased expression level of p-ACC/ACC (Figure 5b), p-AMPK*α*/AMPK*α* (Figure 5c), and p-AMPK*β*1/AMPK*β*1/2 (Figure 5f) was correlated with suppression of PPAR*γ* and C/EBP*α* protein expression (Figure 4e) as well as lipid accumulation in differentiated 3T3-L1 cells (Figure 4a). The results presented in this study correspond with the evidence of the stimulation of AMPK and ACC protein being associated with the inhibition of 3T3-L1 adipocyte differentiation [44]. Interestingly, the densifloral B (**3**) also suppressed the activated Akt (p-Akt), an upstream regulator of the AMPK–ACC signal [45] to reduce the adipocyte formation (Figure 5d).

**Figure 6:**
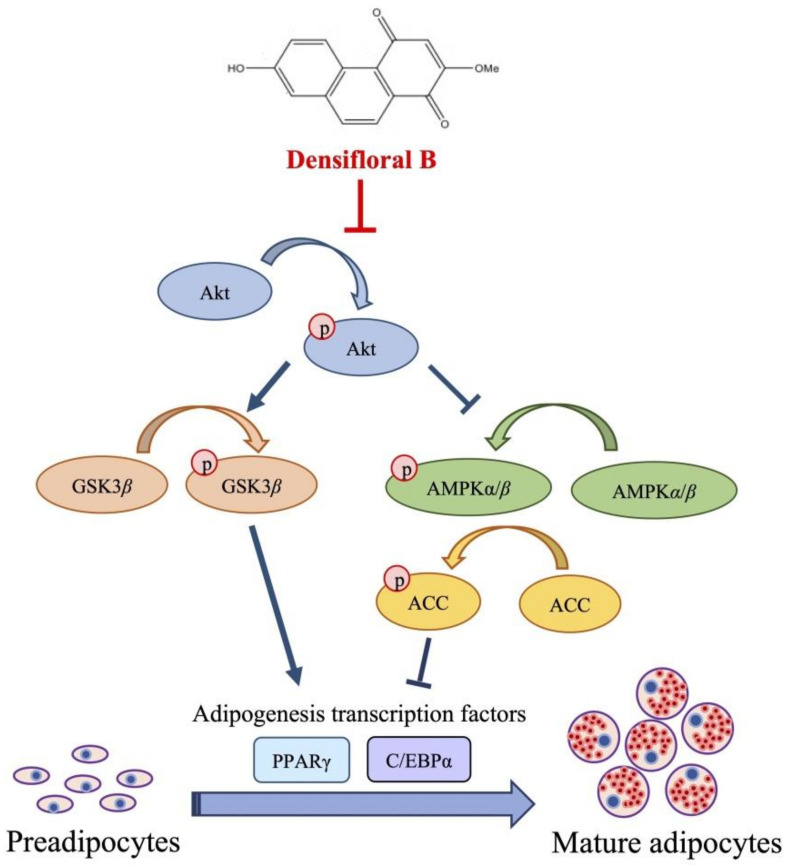
Proposed mechanism of densifloral B (**3**) on inhibition of adipogenesis via modulating Akt-related pathways. Activated Akt (p-Akt) mediates adipocyte differentiation through upregulation of p-GSK3*β* and suppression of AMPK–ACC signal that both trigger the expression of adipogenic transcription factors PPARγ and C/EBP*α*. Therefore, the downregulation of p-Akt moderated by densifloral B (**3**), as a consequence of the restraint of p-GSK3*β* and stimulated AMPK–ACC cascades, effectively inhibits the differentiation of preadipocytes into mature adipocytes.

Due to the expression of adipogenic transcription factors also modulated via Akt/GSK3*β* [46,47,48], the alteration of the p-GSK3*β*/GSK3*β* level was additionally examined in densifloral B-treated 3T3-L1 cells. The phosphorylation by p-Akt results in the inactivation of the GSK3*β* degradation complex following the initiation of targeted gene expression [49]. Surprisingly, the presence of 20 µM densifloral B (**3**) in differentiation medium significantly lessened p-GSK3*β*/GSK3*β* expression level in preadipocyte 3T3-L1 cells at 38–48 h of differentiation time (Figure 5e). Taken together, the present results suggest that densifloral B (**3**) isolated from *D. delacourii* might inhibit adipocyte differentiation via suppression of the Akt-mediating GSK3*β* and AMPK–ACC signals (Figure 6).

## 3. Materials and Methods

### 3.1. General Experimental Procedures

Mass spectra were recorded on a Bruker micro TOF mass spectrometer (ESI-MS). NMR spectra were recorded on a Bruker Avance DPX-300FT-NMR spectrometer or a Bruker Avance III HD 500 NMR spectrometer. Vacuum-liquid column chromatography (VLC) and column chromatography (CC) were performed on silica gel 60 (Merck, Kieselgel 60, 70–320 mesh), silica gel 60 (Merck, Kieselgel 60, 230–400 mesh) (Darmstadt, Germany), and Sephadex LH-20 (25–100 μm, Pharmacia Fine Chemical Co. Ltd.) (Piscataway, NJ, USA). Acarbose was purchased from Fluka Chemical (Buchs, Switzerland). Mouse embryonic preadipocyte 3T3-L1 cells were acquired from the American Type Culture Collection (ATCC, Manassas, VA, USA). Dulbecco’s modified Eagle medium (DMEM), fetal bovine serum (FBS), penicillin/streptomycin, and l-glutamine were obtained from Gibco (Gaithersburg, MA, USA). Yeast alpha-glucosidase enzyme, *p*-nitrophenol-*α*-D-glucopyranoside, isobutylmethylxanthine, dexamethasone, MTT (methyl-thiazolyl-diphenyl-tetrazolium bromide), Hoechst33342, propidium iodide, dimethylsulfoxide (DMSO), and oil red O were purchased from Sigma-Aldrich (St. Louis, MO, USA). Insulin was obtained from Himedia (Mumbai, India). The radio-immunoprecipitation assay (RIPA) buffer, chemiluminescent substrates, and the bicinchoninic acid (BCA) protein assay kit were from Thermo Scientific (Rockford, IL, USA), and the nitrocellulose membranes were from Bio-Rad Laboratories (Hercules, CA, USA). All primary and secondary antibodies were purchased from Cell Signaling Technology (Danvers, MA, USA). Oxyresveratrol was provided by Prof. Kittisak Likhitwitayawuid.

### 3.2. Plant Material

The whole plant of *Dendrobium delacourii* was purchased from a Chatuchak market in May 2018. Plant identification was performed by Dr. Boonchoo Sritularak. A voucher specimen (BS-Ddela-052561) was deposited at the Department of Pharmacognosy and Pharmaceutical Botany, Faculty of Pharmaceutical Sciences, Chulalongkorn University.

### 3.3. Extraction and Isolation

The dried powder of whole plant *D. delacourii* (3.5 kg) was macerated with MeOH (4 × 15 L), and a methanolic extract (300.6 g) was obtained. This extract was dissolved in water and then partitioned with ethyl acetate (EtOAc) and butanol to give an EtOAc extract (159.7 g), a butanol extract (98.2 g), and an aqueous extract (42.1 g), respectively, after evaporation of the solvent. The EtOAc extract exhibited 85.5% inhibition of α-glucosidase enzyme at 100 µg/mL and also showed 49.0% inhibition of adipocyte differentiation of 3T3-L1 cells at 5 µg/mL, whereas the other extracts were devoid of both activities. Therefore, the EtOAc extract was subjected to further investigation.

The EtOAc extract was separated by vacuum liquid chromatography (silica gel, acetone–hexane, gradient) to give five fractions (A–E). Fraction D (54.2 g) was fractionated on a silica gel column (acetone–hexane, gradient) to give four fractions (DA–DD). Fraction DB (5.4 g) was separated by Sephadex LH-20 (methanol) to yield six fractions (DBA–DBF). Fraction DBB (612.0 mg) was subjected to column chromatography (CC) (silica gel, acetone–hexane, gradient), and then the pure compounds hircinol (**1**) (11.4 mg) and ephemeranthoquinone (**2**) (6.4 mg) were obtained. Densifloral B (**3**) (7.9 mg), moscatin (**4**) (17.1 mg), and 4,9-dimethoxy-2,5-pheneanthrenediol (**5**) (3.6 mg) were obtained from fractions DBD, DBE, and DBF after purification on a silica gel column (acetone–hexane, gradient). Fraction DC (6.1 g) was separated by Sephadex LH-20 (methanol) to yield four fractions (DCA–DCD). The separation of fraction DCA (1.2 g) by CC (silica gel, acetone–hexane, gradient) resulted in gigantol (**6**) (166.5 mg). Fraction DCB (170.9 mg) was separated on a silica gel column (EtOAc-CH_2_Cl_2_, gradient) to yield batatasin III (**7**). Fraction DCD (428.0 mg) was isolated by a silica gel column (acetone–hexane, gradient) to give lusianthridin (**8**). Fraction DD (6.7 g) was separated by Sephadex LH-20 (methanol) to yield three fractions (DDA-DDC). Quantities of 4,4′,7,7′-tetrahydroxy-2,2′-dimethoxy-9,9′,10,10′-tetrahydro-1,1′-biphenanthrene (**9**) (4.7 mg) and phoyunnanin E (**10**) (7.0 mg) were obtained from fraction DDB, and phoyunnanin C (**11**) (6.8 mg) was obtained from fraction DDC after separation in a silica gel column (methanol–CH_2_Cl_2_, gradient).

Hircinol (**1**): yellow amorphous solid; HR-ESI-MS: *m/z* 243.1059 [M + H]^+^ calcd. for C_15_H_15_O_3_, 243.1021, suggesting C_15_H_14_O_3._^1^H NMR (500 MHz, acetone-*d*_6_) δ: 2.59 (4H, m, H_2_-9 and H_2_-10), 3.97 (3H, s, 4-MeO), 6.56 (1H, d, *J* = 2.5 Hz, H-1), 6.60 (1H, d, *J* = 2.5 Hz, H-3), 6.80 (2H, d, *J* = 8.0 Hz, H-6, H-8), 7.06 (1H, t, *J* = 8.0 Hz, H-7); ^13^C NMR (125 MHz, acetone-*d*_6_) δ: 31.6 (C-10), 31.8 (C-9), 57.2 (4-MeO), 99.8 (C-3), 109.8 (C-1), 114.6 (C-4b), 118.2 (C-6), 120.1 (C-8), 128.1 (C-7), 130.4 (C-4a), 141.3 (C-8a), 144.2 (C-10a), 154.7 (C-4), 156.3 (C-5), 158.5 (C-2).

Ephemeranthoquinone (**2**): reddish powder; HR-ESI-MS: *m/z* 279.06185 [M + Na]^+^ calcd. for C_15_H_12_O_4_Na, 279.06333 suggesting C_15_H_12_O_4_. ^1^H NMR (300 MHz, acetone-*d*_6_) δ: 2.62 (2H, m, H_2_-10), 2.69 (2H, m, H_2_-9), 3.85 (3H, s, 2-OMe), 5.98 (1H, s, H-3), 6.76 (2H, m, H-6, H-8), 7.97 (1H, d, *J* = 9.3 Hz, H-5); ^13^C NMR (75 MHz, acetone-*d*_6_) δ: 20.0 (C-10), 27.2 (C-9), 55.7 (2-OMe), 107.4 (C-3), 113.4 (C-5), 114.8 (C-8), 121.2 (C-4b), 132.0 (C-6), 136.1 (C-4a), 136.2 (C-10a), 141.5 (C-8a), 158.6 (C-2), 159.1 (C-7), 180.8 (C-1), 187.2 (C-4).

Densifloral B (**3**): orange powder, HR-ESIMS: *m/z* 277.0473 [M + Na]^+^ calcd. for C_15_H_10_O_4_Na, 277.04768 suggesting C_15_H_10_O_4_. ^1^H NMR (300 MHz, acetone-*d*_6_) δ: 3.92 (3H, s, 2-OMe), 6.20 (1H, s, H-3), 7.31 (1H, d, *J* = 2.4 Hz, H-8), 7.35 (1H, dd, *J* = 2.4, 9.3 Hz, H-6), 8.04 (2H, br s, H-9 and H-10), 9.49 (1H, d, *J* = 9.3 Hz, H-5); ^13^C NMR (75 MHz, acetone-*d*_6_) δ: 55.8 (2-OMe), 109.9 (C-8), 110.9 (C-3), 122.0 (C-5, C-6), 124.2 (C-4b), 127.5 (C-4a), 130.1 (C-10a), 132.3 (C-10), 134.2 (C-9), 139.4 (C-8a), 157.8 (C-7), 160.2 (C-2), 180.5 (C-1), 188.4 (C-4).

Moscatin (**4**): brown amorphous solid, HR-ESIMS: *m/z* 241.0888 [M + H]^+^ calcd. for C_15_H_13_O_3_ 241.0865, suggesting C_15_H_12_O_3_. ^1^H NMR (500 MHz, acetone-*d*_6_) δ: 4.15 (3H, s, 4-OMe), 6.99 (1H, d, *J* = 2.5 Hz, H-3), 7.07 (1H, d, *J* = 2.5 Hz, H-1), 7.10 (1H, dd, *J* = 7.5, 2.0 Hz, H-6), 7.41 (1H, dd, *J* = 7.5, 2.0 Hz, H-8), 7.43 (1H, t, *J* = 7.5 Hz, H-7), 7.49 (1H, d, *J* = 9.0 Hz H-10), 7.63 (1H, d, *J* = 9.0 Hz, H-9); ^13^C NMR (125 MHz, acetone-*d*_6_) δ: 58.6 (4-OMe), 102.5 (C-3), 107.8 (C-1), 113.9 (C-4a), 116.9 (C-6), 119.8 (C-4b), 121.0 (C-8), 126.9 (C-10), 127.4 (C-7), 129.7 (C-9), 135.0 (C-8a), 137.1 (C-10a), 155.2 (C-5), 156.4 (C-2), 157.3 (C-4).

4,9-Dimethoxy-2,5-phenanthrenediol (**5**): brown amorphous solid. HR-ESIMS: at *m/z* 271.1009, [M + H]^+^ calcd for C_16_H_15_O_4_; 271.0970, suggesting C_16_H_14_O_4_. ^1^H NMR (500 MHz, acetone-*d*_6_) δ: 4.03 (3H, s, 9-OMe), 4.11 (3H, s, 4-OMe), 6.81 (1H, d, *J* = 2.5 Hz, H-3), 6.92 (1H, s, H-10), 6.99 (1H, d, *J* = 2.5 Hz, H-1), 7.12 (1H, dd, *J* = 1.5, 7.5 Hz, H-6), 7.43 (1H, t, *J* = 7.5 Hz, H-7), 7.85 (1H, dd, *J* = 1.5, 7.5 Hz, H-8), 8.82 (1H, s, 2-OH), 9.43 (1H, s, 5-OH); ^13^C NMR (125 MHz, acetone-*d*_6_) δ: 55.9 (9-OMe), 58.5 (4-OMe), 100.3 (C-3), 102.8 (C-10), 106.9 (C-1), 110.0 (C-4a), 114.3 (C-8), 117.6 (C-6), 120.9 (C-4b), 127.9 (C-7), 129.2 (C-8a), 137.9 (C-10a), 154.9 (C-9), 155.2 (C-5), 156.3 (C-4), 157.5 (C-2).

Gigantol (**6**): brown amorphous solid. HR-ESIMS: at *m/z* 297.1102, [M + Na]^+^ calculated for C_16_H_18_O_4_Na; 297.1102, suggesting C_16_H_18_O_4_. ^1^H NMR (500 MHz, acetone-*d*_6_) δ: 2.79 (4H, m, H_2_-α, H_2_-α′), 3.69 (3H, s, 3-OMe), 3.77 (3H, s, 3′-OMe), 6.25 (1H, t, *J* = 1.5 Hz, H-4), 6.29 (1H, t, *J* = 1.5 Hz, H-6), 6.32 (1H, t, *J* = 1.5 Hz, H-2), 6.65 (1H, dd, *J* = 8.0, 2.0 Hz, H-6′), 6.73 (1H, d, *J* = 8.0 Hz, H-5′), 6.79 (1H, d, *J* = 2.0 Hz, H-2′); ^13^C NMR (125 MHz, acetone-*d*_6_) δ: 37.9 (C-α′), 38.9 (C-α), 55.2 (3′-OMe), 56.1 (3-OMe), 99.7 (C-4), 106.3 (C-6), 108.9 (C-2), 112.8 (C-5′), 115.5 (C-2′), 121.5 (C-6′), 134.1 (C-1′), 145.1 (C-4′), 145.4 (C-1), 147.9 (C-3′), 159.2 (C-3), 161.8 (C-5).

Batatasin III (**7**): brown amorphous solid. HR-ESIMS: at *m/z* 267.10556, [M + Na]^+^ calculated for C_15_H_16_O_3_Na; 267.099715, suggesting C_15_H_16_O_3_. ^1^H NMR (500 MHz, acetone-*d*_6_) δ: 2.79 (4H, m, H-α, H-α′), 3.70 (3H, s, 3-OMe), 6.23 (1H, t, *J* = 2.0 Hz, H-4), 6.30 (1H, t, *J* = 2.0 Hz, H-2), 6.32 (1H, br t, *J* = 2.0 Hz, H-6), 6.63 (1H, m, H-4′), 6.69 (1H, br d, *J* = 9.0 Hz, H-6′), 6.71 (1H, br d, *J* = 2.4 Hz, H-2′), 7.07 (1H, t, *J* = 8.0 Hz, H-5′); ^13^C NMR (125 MHz, acetone-*d*_6_) δ: 38.2 (C-α′), 38.5 (C-α), 55.2 (3-OMe), 99.8 (C-4), 106.2 (C-2), 108.8 (C-6), 113.6 (C-4′), 116.2 (C-2′), 120.4 (C-6′), 130.0 (C-5′), 144.3 (C-1′), 145.0 (C-1), 158.2 (C-3′), 159.2 (C-3), 161.8 (C-5).

Lusianthridin (**8**): brown amorphous solid. HR-ESIMS: at *m/z* 265.08251, [M + Na]^+^ calculated for C_15_H_14_O_3_Na; 265.084065, suggesting C_15_H_14_O_3_. ^1^H NMR (500 MHz, acetone-*d*_6_) δ: 2.67 (4H, m, H_2_-9 and H_2_-10), 3.72 (3H, s, 2-OMe), 6.37 (1H, d, *J* = 2.5 Hz, H-1), 6.45 (1H, d, *J* = 2.5 Hz, H-3), 6.73 (1H, br d, *J* = 7.5 Hz, H-6), 6.72 (1H, br s, H-8), 8.24 (1H, d *J* = 7.5 Hz, H-5); ^13^C NMR (125 MHz, acetone-*d*_6_) δ: 30.6 (C-9), 31.3 (C-10), 55.2 (2-OMe), 101.5 (C-3), 105.8 (C-1), 113.4 (C-6), 115.0 (C-8), 115.7 (C-4a), 125.8 (C-4b), 129.8 (C-5), 139.7 (C-8a), 141.3 (C-10a), 155.7 (C-4), 155.8 (C-7), 159.1 (C-2).

4,4′,7,7′-Tetrahydroxy-2,2′-dimethoxy-9,9′,10,10′-tetrahydro-1,1′-biphenanthrene (**9**): yellow amorphous powder, HR-ESIMS: at *m/z* 505.1630, [M + Na]^+^ calculated for C_30_H_26_O_6_Na; 505.1627 suggesting C_30_H_26_O_6._ ^1^H NMR (300 MHz, acetone-*d*_6_) δ: 2.31 (4H, m, H_2_-10, H_2_-10′), 2.51 (4H, m, H_2_-9, H_2_-9′), 3.60 (6H, s, 2-OMe, 2′-OMe), 6.57 (2H, s, H-3, H-3′), 6.65 (2H, d, *J* = 2.4 Hz, H-8, H-8′), 6.69 (2H, dd, *J* = 8.4, 2.4 Hz, H-6, H-6′), 8.25 (2H, d, *J* = 8.4 Hz, H-5, H-5′), 8.08 (2H, s, 7-OH, 7′-OH), 8.41 (2H, s, 4-OH, 4′-OH); ^13^C NMR (75 MHz, acetone-*d*_6_) δ: 27.0 (C-10, C-10′), 29.7 (C-9, C-9′), 54.7 (2-OMe, 2′-OMe), 98.3 (C-3, C-3′), 112.5 (C-6, C-6′), 113.8 (C-8, C-8′), 114.6 (C-4a, C-4a′), 116.5 (C-1, C-1′), 125.5 (C-4b, C-4b′), 129.3 (C-5, C-5′), 139.3 (C-8a, C-8a′), 139.7 (C-10a, C-10a′), 154.0 (C-4, C-4′), 155.1 (C-7, C-7′), 156.4 (C-2, C-2′).

Phoyunnanin E (**10**): amorphous powder, HR-ESIMS: at *m/z* 505.1628, [M + Na]^+^ calculated for C_30_H_26_O_6_Na; 505.1627, suggesting C_30_H_26_O_6_. ^1^H NMR (500 MHz, acetone-*d*_6_) δ: 2.60 (4H, m, H_2_-9 and H_2_-10), 2.67 (4H, m, H_2_-9′, H_2_-10′), 3.71 (3H, s, 2-OMe), 3.73 (3H, s, 2′-OMe), 6.37 (1H, d, *J* = 2.5 Hz, H-1′), 6.42 (1H, d, *J* = 2.5 Hz, H-3′), 6.62 (1H, dd, *J* = 8.5, 2.5 Hz, H-6′), 6.66 (1H, s, H-3), 6.67 (1H, d, *J* = 2.5 Hz, H-8′), 6.69 (1H, d, *J* = 2.5 Hz, H-8), 6.71 (1H, dd, *J* = 2.5, 9.0 Hz, H-6), 8.25 (1H, d, *J* = 8.5 Hz, H-5′), 8.27 (1H, d, *J* = 9.0 Hz, H-5); ^13^C NMR (125 MHz, acetone-*d*_6_) δ: 23.8 (C-10), 30.7 (C-9′, C-9), 31.3 (C-10′), 55.3 (2′-OMe), 56.0 (2-OMe), 100.8 (C-3), 101.6 (C-3′), 106.0 (C-1′), 112.6 (C-6′), 113.6 (C-6), 114.2 (C-8′), 115.0 (C-8), 115.5 (C-4a′), 115.7 (C-4a), 125.6 (C-4b), 127.6 (C-4b′), 129.8 (C-5′), 130.2 (C-5), 133.9 (C-1), 134.0 (C-10a), 139.7 (C-8a′), 139.8 (C-8a), 141.6 (C-10a′), 152.0 (C-2), 152.5 (C-4), 156.1 (C-4′), 156.4 (C-7), 157.7 (C-7′), 159.6 (C-2′).

Phoyunnanin C (**11**): amorphous powder, HR-ESIMS: at *m/z* 505.1635, [M + Na]^+^ calculated for C_30_H_26_O_6_Na; 505.1627, suggesting C_30_H_26_O_6_. ^1^H NMR (300 MHz, acetone-*d*_6_) δ: 2.51 (2H, m, H_2_-10), 2.53 (2H, m, H_2_-9), 2.73 (4H, m, H_2_-9′, H_2_-10′), 3.64 (3H, s, 2-OMe), 3.72 (3H, s, 2′-OMe), 6.38 (2H, br s, H-1′, H-3′), 6.57 (1H, s, H-3), 6.66 (1H, d, *J* = 2.7 Hz, H-8), 6.69 (1H, dd, *J* = 8.4, 2.7 Hz, H-6), 6.76 (1H, s, H-8′), 8.08 (1H, s, H-5′), 8.23 (1H, d, *J* = 8.4 Hz, H-5); ^13^C NMR (75 MHz, acetone-*d*_6_) δ: 27.5 (C-10), 29.6 (C-9, C-9′), 30.7 (C-10′), 54.4 (2-OMe), 54.8 (2′-OMe), 98.5 (C-3), 100.7 (C-3′), 105.1 (C-1′), 112.5 (C-6), 113.8 (C-8), 114.3 (C-8′), 114.9 (C-4a), 115.1 (C-4a′), 117.4 (C-1), 121.7 (C-6′), 124.8 (C-4b′), 125.3 (C-4b), 129.3 (C-5), 131.7 (C-5′), 137.7 (C-8a′), 139.2 (C-8a), 140.1 (C-10a), 140.5 (C-10a′), 152.8 (C-7′), 154.3 (C-4), 155.1 (C-4′), 155.2 (C-7), 156.6 (C-2), 158.3 (C-2′).

### 3.4. Assay for α-Glucosidase Inhibitory Activity

The assay was based on the inhibition in the sample of *α*-glucosidase enzyme, which can release *p*-nitrophenol (PNP) from *p*-nitrophenyl-*α*-D-glucoside (PNPG) by hydrolysis [48]. In this assay, acarbose was used as the positive control. For IC_50_ determination, twofold serial dilution was performed for each sample. Each experiment was accomplished in triplicate. Data are expressed as mean ± SD.

The kinetic study of enzyme inhibition was analyzed by the double reciprocal Lineweaver–Burk plot (1/V versus 1/[S]). The experiment was carried out by performing various concentrations of substrate *p*-nitrophenol-*α*-D-glucopyranoside (0.25, 0.5, 1.0, 2.0 mM) in the absence or presence of compound (**10**) (12 and 22 μM) and compound (**11**) (12 and 24 μM). The reaction was monitored every 5 min for a total time of 25 min and measured at 405 nm by a microplate reader. Each experiment was performed in triplicate. The K*_i_* value was estimated by constructing a secondary plot which is plotted by the slopes of the double-reciprocal lines versus inhibitor concentration.

### 3.5. Assay for Anti-Adipogenic Activity

#### 3.5.1. Cell Culture and Adipocyte Differentiation

Mouse embryonic preadipocyte 3T3-L1 cells were cultured in DMEM containing 10% FBS, 100 units/mL of penicillin/streptomycin, and 2 mmol/L of l-glutamine under humidified conditions of 5% CO_2_ at 37 °C until 70–80% confluence was reached. For differentiation into adipocyte, preadipocyte 3T3-L1 cells were incubated with differentiation media composed of 10% FBS, 0.5 mM isobutylmethylxanthine, 1 μM dexamethasone, and 5 µg/mL insulin in DMEM with or without test compound for 2 days. Then, the differentiation media was replaced with culture media containing 5 µg/mL of insulin. After further incubation for 2 days, the cells were maintained in complete DMEM, which was changed every 2 days until adipocytes containing lipid droplets were observed under microscope [40].

#### 3.5.2. Determination of Cytotoxicity

To evaluate the effect of *D. delacourii* extracts on cell viability, 3T3-L1 preadipocytes were seeded into 96-well plates at density of 2 × 10^3^ cells/well and allowed to attach overnight at 37 °C. Then, the cells were further cultured with extracts (5 μg/mL), compounds (20 μM), or left untreated for 48 h before adding of 0.45 mg/mL MTT solution to assess cell viability. After incubation for 3 h at 37 °C and kept from light, the optical density (OD) of the purple formazan product dissolved in DMSO was measured at 570 nm using a microplate reader (Anthros, Durham, NC, USA). The relative OD ratio of treated to non-treated cells was presented as percentage cell viability [50].

The cytotoxicity of *D. delacourii* extracts was confirmed via cell death detection using costaining of Hoechst33342 and propidium iodide. After 48 h incubation with indicated treatment, the cells were further incubated with nuclear staining solution containing 2 μg/mL of Hoechst33342 and 1 μg/mL of propidium iodide for 30 min. The mode of cell death was observed under a fluorescence microscope (Olympus IX51 with DP70, Olympus Corp., Shinjuku-ku, Tokyo, Japan).

#### 3.5.3. Quantification of Cellular Lipid Content Using Oil Red O Staining

The lipid droplets presenting in differentiated adipocytes were detected via oil red O staining. After the differentiation process, 3T3-L1 cells were fixed with 10% formalin for 45 min and further incubated with oil red O solution at room temperature for 1 h. After washing with 60% isopropanol for three times, oil red O-stained cells were captured using a Nikon Ts2 inverted optical microscope (Tokyo, Japan). For quantification, cellular oil red O was extracted using absolute isopropanol for measurement of OD at 570 nm by microplate reader (Anthros, Durham, NC, USA) [51]. The percentage of oil red O staining was calculated relative to the total protein content determined by BCA assay [52].

#### 3.5.4. Western Blot Analysis

After the indicated treatment, 3T3-L1 cells were washed with phosphate-buffered saline (PBS, pH 7.4), then the cell membranes were broken using RIPA buffer supplemented with a protease inhibitor cocktail. After incubation on ice for 45 min, the cell lysates were centrifuged at 12,000 rpm at 4 °C for 15 min to collect the clear supernatant containing cellular protein, which was measured for total protein content using a BCA assay kit. The total protein (30 µg) from each sample was loaded and separated onto 10% sodium dodecyl sulfate-polyacrylamide gel electrophoresis (SDS-PAGE). Subsequently, the separated proteins were transferred onto nitrocellulose membranes, which were blocked with 5% skim milk in TBST buffer (Tris-buffered saline with Tween 20, pH 7.2) and further immunoblotted with primary antibodies against p-Akt (Thr308), Akt, p-GSK3*β* (Ser9), GSK3*β*, p-AMPK*α* (Thr172), AMPK*α*, p-AMPK*β*1 (Ser128), AMPK*β*1/2, p-ACC (Ser79), ACC, PPAR*γ*, C/EBP*α*, and *β*-actin at 4 °C overnight. Before immersion in horseradish peroxidase (HRP)-linked secondary antibody at room temperature for 2 h, the membranes were washed with TBST for 7 min, three times. The reactive protein signals exposed with chemiluminescent substrates were captured and quantified using Chemiluminescent ImageQuant LAS 4000 (GE Healthcare Bio-Sciences AB, Björkgatan, Uppsala, Sweden).

### 3.6. Statistical Analysis

All data are expressed as means ± standard deviation (SD) obtained from three independent experiments. Statistical analysis was performed using GraphPad Prism 8.0.2 (GraphPad Software Inc., San Diego, CA, USA) with one-way ANOVA. Differences with *p* value < 0.05 were considered to be statistically significant.

## 4. Conclusions

In this study, 11 compounds were isolated from the ethyl acetate extract of *D. delacourii*. Two dimeric phenanthrene derivatives, phoyunnanin E (**10**) and phoyunnanin C (**11**), revealed the most potent *α*-glucosidase inhibition when compared with a positive control, acarbose. An enzyme kinetic study performed on them indicated non-competitive inhibitors. Regarding anti-adipogenic activity, densifloral B (**3**) showed the most potent activity when compared with a positive control, oxyresveratrol. The anti-adipogenic properties of densifloral B (**3**) were attributed to the downregulation of PPAR*γ* and C/EBP*α* expression through the modulation of Akt-related pathways including the Akt/GSK3*β* and Akt/AMPK–ACC signals. The findings obtained from this study demonstrate the evaluation of *α*-glucosidase inhibitory activity and anti-adipogenic effect of the *Dendrobium delacourii* plant, which can be used for the management of diabetes and obesity.

## Figures and Tables

**Table 1:** *α*-Glucosidase inhibitory activity of compounds **1**–**11** from the EtOAc extract.

Compounds	IC_50_ (μM)
Hircinol (**1**)	NA
Ephemeranthoquinone (**2**)	NA
Densifloral B (**3**)	NA
Moscatin (**4**)	390.1 ± 9.8
4,9-Dimethoxy-2,5-phenanthrenediol (**5**)	NA
Gigantol (**6**)	191.3 ± 6.8
Batatasin III (**7**)	NA
Lusianthridin (**8**)	195.4 ± 9.6
4,4′,7,7′-Tetrahydroxy-2,2′-dimethoxy-9,9′,10,10′ -tetrahydro-1,1′-biphenanthrene (**9**)	18.4 ± 3.4
Phoyunnanin E (**10**)	8.9 ± 0.8
Phoyunnanin C (**11**)	12.6 ± 0.9
Acarbose	514.4 ± 9.2

NA = no inhibitory activity.

**Table 2:** Kinetic parameters of *α*-glucosidase inhibition in the presence of phoyunnanin E (**10**) and phoyunnanin C (**11**).

Inhibitors	Dose (μM)	V*_max_* ∆OD_/_min	K*_m_* (mM)	K*_i_* (μM)
None	-	0.10	1.22	
**10**	22	0.024	1.22	5.89
	12	0.049	1.21	
**11**	24	0.023	1.21	5.97
	12	0.049	1.21	
Acarbose	930	0.11	6.47	190.57
	465	0.10	4.17	

V*_max_*, maximum rate of velocity; K*_m_*, Michaelis constant; K*_i_*, inhibitor constant.

## Data Availability

All data presented in this study are available in the article.

## Sample Availability

Samples of the compounds are not available from authors.
